# Supplementary material for: Australian Consumers’ Attitudes towards Sustainable Diet Practices Regarding Food Waste, Food Processing, and the Health Aspects of Diet: A Cross Sectional Survey
Source: Int J Environ Res Public Health. 2023 Feb 1;20(3):2633. doi: 10.3390/ijerph20032633 (PMC9914991; doi:10.3390/ijerph20032633)
Supplement: Supplementary file 1 [file ijerph-20-02633-s001.zip › ijerph-2140864-SI.pdf]

Table S1: **Descriptive analysis results from the demographic characteristics of those who pay attention to sustainable household food waste and packaging disposal, health and food processing level when making food choices.**

		**Health attention**			**Processed food attention in diet**			**Concern for sustainable food packaging disposal and food waste**		
		No n(%)	yes n(%)	Chi sq p value	No n(%)	Yes n(%)	Chi sq p value	No n(%)	Yes n(%)	Chi sq p value
**Total**		77%	23%		72%	28%		63%	37%	
**Gender**	Male	204(49%)	67(54%)	0.639	192(50%)	79(52%)	0.100	181(53%)	90(46%)	0.088
	Female	207(50%)	56(45%)		192(50%)	71(46%)		159(47%)	104(53%)	
	Non-binary/fluid/trans	3(<1%)	1(<1%)		1(<1%)	3(2%)		1(<1%)	3(2%)	
	Prefer not to say	1(0%)	1(0%)		1(0%)	1(0%)		1(0%)	1(0%)	
**Age 3 categories**	18-34 years	114(28%)	27(22%)	0.447	110(28%)	31(20%)	0.139	84(25%)	57(29%)	0.264
	35-54 years	135(32%)	43(35%)		122(32%)	56(37%)		109(32%)	69(35%)	
	55 and over years	166(40%)	54(44%)		154(40%)	66(43%)		148(43%)	72(36%)	
**BMI**	Healthy weight	144(38%)	48(45%)	0.39	140(39%)	52(39%)	0.446	121(39%)	71(40%)	0.023
	Overweight	122(32%)	32(30%)		107(30%)	47(35%)		87(28%)	67(38%)	
	Obese	115(30%)	27(25%)		108(30%)	34(26%)		102(33%)	40(22%)	
**Education -**	≤Year 12	153(37%)	34(28%)	0.064	151(39%)	36(24%)	<0.001	137(40%)	50(25%)	<0.001
	>Year 12	261(63%)	88(72%)		234(61%)	115(76%)		202(60%)	147(75%)	
**Occupation**	Not work in an office	158(45%)	42(42%)	0.511	143(45%)	57(44%)	0.871	128(46%)	72(43%)	0.542
	Work in an office	191(55%)	59(58%)		177(55%)	73(56%)		153(54%)	97(57%)	
**Income**	<AUD$50K	203(49%)	47(38%)	<0.001	189(49%)	61(40%)	0.028	168(49%)	82(41%)	0.147
	AUD$50-99K	87(21%)	17(14%)		78(20%)	26(17%)		66(19%)	38(19%)	
	>+AUD$100	126(30%)	60(48%)		120(31%)	66(43%)		108(32%)	78(39%)	
